# TLR4 polymorphisms may increase susceptibility to periodontitis in Pg-positive individuals

**DOI:** 10.7717/peerj.7828

**Published:** 2019-11-21

**Authors:** Wenjing Li, Xiaojing Cao, Lu He, Huanxin Meng, Bingtao Yang, Yanting Liao

**Affiliations:** Department of Periodontology, National Engineering Laboratory for Digital and Material Technology of Stomatology, Beijing Key Laboratory of Digital Stomatology, Peking University School and Hospital of Stomatology, Beijing, China

**Keywords:** Chronic periodontitis, Toll-like receptor 4, Pg, SNP

## Abstract

**Objective:**

To investigate the correlation between the single nucleotide polymorphisms (SNPs) in the toll-like receptor 4 (TLR4) gene and the susceptibility to chronic periodontitis.

**Design:**

241 Chinese subjects from the cohort of Beijing Shijingshan Community were recruited. Buccal swab samples, the whole unstimulated saliva and periodontal clinical parameters were collected. Human DNA extracted from buccal swab samples were used for genotyping eight SNPs of the TLR4 gene (rs11536889, rs1927906, rs1927911, rs2149356, rs4986790, rs4986791, rs2737190, rs787384) by the Sequenom MassARRAY system. *Porphyromonas gingivalis* (*P. gingivalis*) was detected from the deposition of the whole unstimulated saliva through polymerase chain reaction (PCR) method based on 16S rRNA. The correlation between SNPs of TLR4 and chronic periodontitis susceptibility in the whole subjects and the patients detected with *P. gingivalis* was investigated.

**Results:**

The variants of rs4986790 and rs4986791 were not found in 241 Chinese subjects. Moreover, there was no significant difference in the distribution of theother6 SNPs of TLR4 between groups of none/mild -periodontitis and moderate/severe-periodontitis subjects. When combined with *P. gingivalis* infection, rs1927911 (TT/CC+CT), rs2149356 (TT/GG+GT) and rs2737190 (GG/AA+AG) were independent risk factors of chronic periodontitis.

**Conclusion:**

Three SNPs of TLR4, i.e., rs1927911 (TT/CC+CT), rs2149356 (TT/GG+GT) and rs2737190 (GG/AA+AG), were associated with moderate/severe chronic periodontitis in Chinese population infected with *P. gingivalis*. *P. gingivalis*, which interacted with TLR4 gene plays an important role in the pathogenesis of periodontitis.

## Introduction

Periodontal diseases are initiated by microorganisms in the subgingival biofilm, of which *Porphyromonas gingivalis* (*P. gingivalis,* Pg) was revealed to be the most prevalent gram-negative bacteria among patients with chronic periodontitis when compared with healthy controls (Feng et al., 2014; Van Winkelhoff et al., 2002). What’s more, its lipopolysaccharide (LPS) as well as other virulence factors could cause significant inflammatory response (Lamont & Jenkinson, 1998). *P. gingivalis* mainly colonizes at subgingival sites, especially in deep pockets, of patients with periodontitis (Farias et al., 2012). Thus, its prevalence was much higher and its amount was far higher in moderate and severe periodontitis patients than the mild periodontitis patients or periodontally healthy subjects (Feng et al., 2009). Several studies showed that detection of *P. gingivalis* in saliva samples matched with that in subgingival samples of periodontitis patients (Feng et al., 2009; He et al., 2012).

However, as one of the major periodontal pathogens with convincing evidence, the presence of *P. gingivalis* doesn’t mean that the individual suffers from moderate or severe periodontitis. In fact, *P. gingivalis* can also be detected in periodontal healthy controls (Feng et al., 2014; Van Winkelhoff et al., 2002). Therefore, there are likely to be other factors affecting the susceptibility of periodontitis, which should not be ignored.

According to the former studies, genetic factors are important in the host response and individuals’ susceptibility to periodontitis (Genco & Borgnakke, 2013). Single nucleotide polymorphisms (SNPs), as the most common form of gene polymorphism, can indicate susceptibility. In recent years, the association between toll-like receptors 4 (TLR4) SNPs and periodontitis has been investigated extensively. However, results on the relationship of TLR4 SNPs and periodontitis were inconsistent, especially in patients with chronic periodontitis (Chrzeszczyk, Konopka & Zietek, 2015; Song, Kim & Lee, 2013).

TLR4 plays a significant role in the host’s susceptibility for periodontitis (Agnese et al., 2002). It belongs to the pattern recognition receptors (PRRs) family and was found on the surface of immune competent cells. PRRs specifically recognize pathogen-associated molecular patterns (PAMPs) of bacterial wall components such as lipoprotein and LPS (Kay, Scotland & Whiteford, 2014). Because of PRRs, *P. gingivalis* and its LPS can trigger the host immune system through, which will then induce an inflammatory cascade leading to periodontal destruction (Fukusaki et al., 2007). The binding of *P. gingivalis* LPS to TLR4 on human gingival fibroblasts (HGFs) can activate various second messenger systems, such as the nuclear factor κB (NF-κB) pathway. These processes are closely associated with the destruction of periodontal tissue (Wang et al., 2000).

Missense mutation of TLR4 gene can lead to hypo-responsiveness to LPS stimulation in mice, which can make them vulnerable under Gram-negative bacterial infection (Poltorak et al., 1998). At present, the missense mutations in TLR4, rs4986790 (Asp299Gly) and rs4986791 (Thr399Ile), were the most commonly observed ones associated with periodontitis in Caucasian population (Chrzeszczyk, Konopka & Zietek, 2015; Ozturk & Vieira, 2009). Nevertheless, these two mutations were rarely found in Asian patients (Fukusaki et al., 2007; Zhu et al., 2008), which may indicate that gene polymorphisms of TLR4 were highly race-related. Hence, more studies in different populations need to be conducted.

Pathogenesis of periodontitis involves a complex interaction between bacterial infection and susceptible hosts. At the same time, such process of interaction is also modified by environmental and acquired factors (Genco & Borgnakke, 2013). Since Kornman et al. (1997) firstly demonstrated a periodontitis-associated genotype of the polymorphic interleukin-1 (IL-1) gene cluster, numerous studies reported positive or negative relationships among different populations based on race, range of age and disease classification (Karimbux et al., 2012). Meisel et al. (2004) found that smokers bearing the genotype-positive IL-1 allele had an enhanced risk for periodontitis, while the IL-1 genetic polymorphism had no influence on non-smokers. This finding proved the gene-environmental interaction. Furthermore, the genetic impact on the individual susceptibility and severity of periodontitis might be mediated by immune response against bacterial stimulus. Thus, we suppose that gene polymorphisms of TLR4 may also play a crucial role in increasing the susceptibility of periodontitis when patients are suffering from quite a few pathogens.

However, only a few studies have investigated the association between SNPs and periodontitis simultaneously considering keystone pathogens up to date (Gursoy et al., 2016; Laine et al., 2013; Sellers et al., 2016). Recently, two studies in Caucasian population observed the correlation of rs4986790 and the presence of *P. gingivalis* with respect to periodontitis. (Gursoy et al., 2016; Sellers et al., 2016) But the results were controversial. Hence, our hypothesis is that there would be a potential relationship between SNPs of TLR4 and moderate/severe periodontitis with *P. gingivalis* infection. Therefore, eight SNPs (rs11536889, rs1927906, rs1927911, rs2149356, rs4986790, rs4986791, rs2737190, rs787384) of TLR4 were selected and analyzed as the target SNPs of this study. *P. gingivalis* detection was also conducted. The combined bacterial-gene status would further illustrate the complex pathogenesis of periodontitis.

## Material and Methods

### Subjects

Two hundred and forty-one Chinese subjects from the cohort of Beijing Shijingshan Community were recruited. All subjects were under regular care and medical examination at Beijing Hypertension Prevention and Management Institution. The protocol was approved by the Ethics Committee, Medical Science Center of Peking University.

All subjects were Chinese, over 40 years old and didn’t have any other infectious disease except for periodontitis. Individuals with the following conditions were excluded. Patients who had less than 15 teeth, or was ever diagnosed with aggressive periodontitis previously. Patients who received periodontal treatment in less than 6 months or took systemic antibiotics continuously for over a week within 3 months. Patients who had any main adverse cardiac and cerebral events (MACCE) in less than 6 months. Also, patients who were diagnosed with systemic diseases except for diabetes and hypertension.

Informed consents were obtained from all participants upon recruiting. The informed consent is mainly concerned with informing patients of the need for a detailed periodontal examination and obtaining biological samples that may be used for genetic testing. The samples collected from subjects are only used for scientific research and analysis, and do not involve patient privacy exposure.

### Examination

The comprehensive general examination was at the beginning, a comprehensive examination was conducted for every subjects, including height, weight, waist circumference, hip circumference, blood pressure (BP), electrocardiograph (ECG), routine urine test, and blood test.

Then, all selected subjects received full mouth periodontal examination by one skilled calibrated periodontist with a Williams periodontal probe. Probing depth (PD), bleeding index (BI) at 6 sites per tooth, attachment loss (AL) at 2 sites (mesial-buccal and distal-lingual) per tooth, as well as the number of missing teeth were recorded. Percentage of bleeding on probing (BOP%) was also calculated.

Based on the criteria defined by the Centers for Disease Control and American Academy of Periodontology (CDC-AAP) in 1999 (Page & Eke, 2007), subjects were divided into two groups according to their clinical periodontal index. Moderate/severe chronic periodontitis (msCP) group was defined by patients with ≥2 interproximal sites with AL ≥4 mm (not on the same tooth), or ≥2 interproximal sites with PD ≥5 mm (not on the same tooth). According to the literature (Eke et al., 2012), no periodontitis was defined as “No evidence of mild, moderate, or severe periodontitis. Mild periodontitis was defined as “≥2 interproximal sites with AL ≥ 3 mm, and ≥2 interproximal sites with PD ≥4 mm (not on same tooth) or one site with PD ≥5 mm”. The no periodontitis subjects and mild periodontitis patients were merged into one group which named nmCP group.

### *P. gingivalis* detection in Saliva

Unstimulated whole saliva was collected before any periodontal examination between 8 am and 10 am. Subjects were instructed to refrain from eating, drinking or using oral hygiene products for at least 1 h before the collection. After the subjects rinsed with water, they were seated in rest for 10 min letting saliva drop naturally flowing down into the sterile cup. The saliva was then transferred into the 1.5 ml sterilized Eppendorf (EP) tube and the tube were placed into an ice box. The samples were processed within 4 h as the following day during the investigation. Samples were centrifuged at 10,000× g for 10 min, and then the deposit was washed 5 times with 500 µl TE buffer (10 mmol/L Tris-HCl, pH 7.6, 1 mmol/L EDTA) before stored at −20 °C.

Genomic DNA was extracted using a commercial bacteria DNA mini kit (Watson Biotechnologies, Shanghai, P.R. China) following the manufacturer’s instruction. Polymerase Chain Reaction (PCR) for *P. gingivalis* 16s rDNA was then conducted following the well-established procedures (Ashimoto et al., 1996; Feng et al., 2014). Specific primer sequences of *P. gingivalis* used in PCR were shown in Table 1.

### Human gene sequencing

Buccal swab samples were collected and stored at −20 °C immediately after extraction, then used for DNA extraction with TIANamp swab DNA Kit (TIANGEN BIOTECHNOLOGY, BEIJING, CO., Ltd.), following the instruction within the kit package. Eight SNPs of TLR4 gene (rs11536889, rs1927906, rs1927911, rs2149356, rs4986790, rs4986791, rs2737190, rs787384) were genotyped by the Sequenom Mass ARRAY system (Shanghai Benegene Biotechnology Co. Ltd.), which was based on the MALDI-TOF technology.

**Table 1:** *P. gingivalisprimer* sequences and amplified fragment length.

	Primer sequence (5′–3′)	Amplified Fragment Length (bp)
*P. gingivalis*	Upstream: AGG CAG CTT GCC ATA CTG CG	404
	Downstream: ACT GTT AGC AAC TAC CGA TGT	

### Statistical analysis

The R-program was used for statistical analysis. Demography characteristics between nmCP group and msCP group were analyzed by independent sample *T*-test (normal distribution) or Wilcoxon rank sum test (non-normal distribution). Differences in genotype distribution between groups was assessed by the chi-square test. A *P*-value of <0.05 was considered statistically significant. Logistic regression analysis was carried out for individual genotypes, *P. gingivalis* infection and their interaction with other risk factors adjusted, such as age, gender, diabetes, hypertension and smoking to determine their influence on the severity of chronic periodontitis (msCP/nmCP).

## Results

### Demographic and clinical characteristics of all subjects

Demography characteristics of msCP and nmCP patients were shown in Table 2. It is shown that the msCP patients had a higher mean age and percentage of smoking than that of the nmCP patients, both with statistical significance. Though the msCP group had a higher percentage of diabetes, the percentage of diabetes and hypertension showed no significant difference between two groups. Periodontal clinical indices including mean PD, AL, BI and BOP% were also statistically higher in msCP group comparing with those of the nmCP group (Table 2). The percentage of *P. gingivalis* detection was higher in msCP than that in nmCP group, however, without statistical difference (Table 2).

**Table 2:** Demographic and clinical characteristics of two groups.

Variables	nmCP (*n* = 58)	msCP (*n* = 183)	*P*-values
Age (years)	54.16 ± 8.81	60.80 ± 9.19	<0.001
Male gender	21 (36.2%)	92 (50.3%)	0.061
Diabetes melitus	21 (36.2%)	82 (44.8%)	0.249
Hypertension	38 (65.5%)	110 (60.1%)	0.461
BMI	24.58 ± 3.14	24.62 ± 3.24	0.525
Smoking	1 (1.7%)	51 (27.9%)	<0.001
PD (mm)	2.34 ± 0.37	2.82 ± 0.62	<0.001
AL (mm)	1.77 ± 1.06	2.77 ± 1.51	<0.001
BI	1.50 ± 0.43	2.20 ± 0.56	<0.001
BOP (%)	37.56 ± 26.33	58.94 ± 21.67	<0.001
Pg+	35 (60.3%)	129 (70.5%)	0.149

**Notes.**

BMI body mass index PD probing depth AL attachment loss BI bleeding index BOP bleeding on probing Pg+ *P. gingivalis* detectable

### Genotype detection and distribution of TLR4

The mutations of rs4986790 and rs4986791 were not found in the whole subjects. There was no significant difference in the distribution of the other 6 detected SNPs of TLR4 between nmCP and msCP patients (Table 3). In the subgroup of 164 patients detected with *P. gingivalis* infection, the distribution of genotypes of rs1927911, rs2149356 and rs2737190 differed significantly between nmCP and msCP patients (Table 3).

**Table 3:** Comparison on genotype distribution of TLR4 SNPs between nmCP and msCP.

SNPs	Whole group (*n* = 241)		*P*-value	Pg + group (*n* = 164)		*P*-value
Genotype	nmCP [*n* (%)]	msCP [*n* (%)]		nmCP [*n* (%)]	msCP [*n* (%)]	
rs11536889			0.944			0.974
GG	32 (55.17%)	100 (54.64%)		21 (60.00%)	77 (59.69%)	
CC + CG	26 (44.83%)	83 (45.36%)		14 (40.00%)	52 (40.31%)	
rs1927906			0.902			0.247
GG + AG	7 (12.07%)	21 (11.48%)		6 (17.14%)	13 (10.08%)	
AA	51 (87.93%)	162 (88.52%)		29 (82.86%)	116 (89.92%)	
rs1927911			0.126			0.007^*^
TT	12 (20.69%)	23 (12.57%)		11 (31.43%)	16 (12.40%)	
CC + CT	46 (79.31%)	160 (87.43%)		24 (68.57%)	113 (87.60%)	
rs2149356			0.098			0.013^*^
TT	12 (20.69%)	22 (12.02%)		10 (28.57%)	15 (11.63%)	
GG + GT	46 (79.31%)	161 (87.98%)		25 (71.43%)	114 (88.37%)	
rs2737190			0.126			0.007^*^
GG	12 (20.69%)	23 (12.57%)		11 (31.43%)	16 (12.40%)	
AA + AG	46 (79.31%)	160 (87.43%)		24 (68.57%)	113 (87.60%)	
rs7873784			0.076			0.054
CC	1 (1.75%)	0 (0.00%)		1 (2.94%)	0 (0.00%)	
GG + CG	56 (98.25%)	179 (100.00%)		33 (97.06%)	125 (100.00%)	

**Notes.**

* *p* < 0.05.

Pg+ *P. gingivalis* detectable

### Logistic regression analysis for the detected SNPs

In patients infected with *P. gingivalis*, age, gender, diabetes, hypertension, smoking were adjusted. As is shown by the result, there was an association between three SNPs, rs1927911 (TT/CC+CT, OR = 7.06, *p* < 0.001), rs2149356 (TT/GG+GT, OR = 7.60, *p* < 0.001), rs2737190 (GG/AA+AG, OR = 7.04, *p* < 0.001) and moderate/severe chronic periodontitis respectively (Table 4).

**Table 4:** Logistic regression analysis for TLR4 SNPs distribution in patients infected with *P. gingivalis*.

SNPs	AOR	95% CI	*P*-value
rs11536889			0.332
GG	1		
CC+CG	7.429	0.931–59.291	
rs1927906			0.514
GG+AG	1		
AA	4.462	1.180–16.871	
rs1927911			<0.001
TT	1		
CC+CT	7.06	2.03–24.57	
rs2149356			<0.001
TT	1		
GG+GT	7.6	2.17–26.61	
rs2737190			<0.001
GG	1		
AA+AG	7.04	2.03–24.57	
rs7873784			0.225
CC	1		
GG+CG	2.04	0.39–2.97	

**Notes.**

AOR adjusted odds ratio CI confidence interval

# Adjusted for age, gender, diabetes, hypertension and smoking.

For the whole subjects, as shown in Table 5, only the interaction of *P. gingivalis* infection and SNPs, rs1927911 (TT/CC+CT, AOR = 7.20, *P* = 0.004), rs2149356 (TT/GG+GT, AOR = 8.19, *P* = 0.002), rs2737190 (GG/AA+AG, AOR = 7.04, *P* = 0.004), was associated with msCP. That is to say, *P. gingivalis* alone may not be able to cause disease without presence of susceptible genes, although the adjusted odds ratio (AOR) of *P. gingivalis* infection was 0.11 (*P* = 0.059) when analyzed by combination with rs1927911 and rs2737190 respectively.

**Table 5:** Effect of Pg, SNPs and their interaction on severity of periodontitis among the whole subjects by logistic regression.

Variables	AOR^a^	95% CI	*P*-value
rs1927911	–	–	–
Pg	0.11	0.01–1.09	0.059
Pg ×rs1927911	7.2	1.90–27.23	0.004^*^
Rs2149356	–	–	–
Pg	0.08	0.01–0.89	0.39
Pg ×rs2149356	8.19	2.13–31.53	0.002^*^
rs2737190	–	–	–
Pg	0.11	0.01–1.09	0.059
Pg ×rs2737190	7.2	1.90–27.23	0.004^*^

**Notes.**

* *p* < 0.05.

AOR adjusted odds ratio CI confidence interval

a Adjusted for age, gender, diabetes, hypertension and smoking.

## Discussion

There was no significant difference in the distribution of the 8 SNPs (rs11536889, rs1927906, rs1927911, rs2149356, rs4986790, rs4986791, rs2737190, rs787384) of TLR4 between patients with none/mild chronic periodontitis (nmCP) and moderate/severe chronic periodontitis (msCP) in the study. While among patients obviously infected with *P. gingivalis*, the distribution of rs1927911, rs2149356 and rs2737190 differed significantly between nmCP patients and msCP patients. This indicates that chronic periodontitis originates from the combination of bacterial challenge and susceptible hosts. TLR4 gene mutations were associated with periodontitis.

Our present results showed the rs4986790, rs4986791 gene mutations were not found in northern Chinese. The result of rs4986790 and rs4986791 gene mutation is consistent with those researches on them in Chinese and Japanese (Fukusaki et al., 2007; Zhu et al., 2008), while different from the Caucasian population (Chrzeszczyk, Konopka & Zietek, 2015; Ozturk & Vieira, 2009; Schroder et al., 2005). It proved the hypothesis that the presence of the missense mutation of SNPs was highly race-related (Kutikhin, 2011). On top of that, Erridge C et al. (Schroder et al., 2005) suggested that Asp299Gly (rs4986790) and Thr399Ile (rs4986791) mutations showed no deficit in LPS signaling. Thus, other SNPs instead of rs4986790 or rs4986791 may contribute to the host susceptibility toward periodontitis in Asian population.

It is noteworthy that interaction between *P. gingivalis* and rs1927911, rs2149356 and rs2737190 was demonstrated risk factor for msCP while none of them alone could determine the severity of the disease in our subjects. This proved the complex nature of periodontitis where the interaction between bacterial infection and susceptible host is crucial for the onset of pathological changes in periodontal tissues. To our knowledge, this is the first report of these three SNPs with susceptibility of periodontitis. The SNPs rs1927911, rs2149356 and rs2737190 were located in the intron of TLR4 gene. While they do not directly participate in the transcription process causing amino acid substitutions, like rs4986791, (Ozturk & Vieira, 2009), they may influence the gene expression in other modes. Genetic variations in intron might enhance the proportion of mRNA transcripts, splicing or lead to a reduction in the proper splicing regardless of amino acid substitution (Lamba et al., 2003).

Previous studies showed gene mutations of rs2149356 could be found in most Asian populations, including Japanese (Takano et al., 2012), Korean (Suh et al., 2011), Chinese (Huang et al., 2015; Qing et al., 2013), as well as Europeans and other Caucasians (Rasheed et al., 2016). It appeared to be associated with some diseases with inconsistent results. Rs2149356 was conferred the highest increased risk of normal tension glaucoma (NTG) in Japanese (Takano et al., 2012), while in South Korean it had no association with NTG (Suh et al., 2011). It was found that there was no relationship between rs2149356 mutation and type 2 diabetes mellitus (T2DM) (Huang et al., 2015), while its TT genotype increased the risk of gouty arthritis (GA) among southern Chinese (Qing et al., 2013). The levels of TLR4 mRNA in peripheral blood mononuclear cells (PBMCs) and IL1β in serum were significantly increased in the TT genotype acute GA patients, which indicated that rs2149356 polymorphism might have played a critical role in the gene expression process (Qing et al., 2013). However, the concrete mechanism is unclear. Interestingly, the T-allele increased the risk of gout in the clinically-ascertained European, while it decreased the risk of gout in Polynesians (Rasheed et al., 2016). Furthermore, no evidence for association in non-clinically-ascertained incident gout cases was found (Rasheed et al., 2016). These controversies indicated that race and type of disease may influence the result. Therefore, to confirm its association with periodontitis, more studies among different populations need to be done.

SNPs rs1927911 and rs2737190 were shown to be associated with chronic periodontitis in our study. Rs1927911 had been reported to be associated with T2DM in southern Chinese (Peng et al., 2015). The rs1927911 polymorphism of the TLR4 gene may be a risk factor for atherosclerotic cerebral infarction (ACI) in the Southern Han population of Hunan Province (Song et al., 2015). However, it may not be associated with fasting blood sugar (Song et al., 2015). Also, there was no direct relation between rs1927911 and diabetes among the Germans (Kolz et al., 2008). Individuals carrying the heterozygous genotypes for the rs2737190, rs1927911 had significantly decreased risk of hepatocellular carcinoma comparing with those carrying wild-type homozygous genotypes, indicating the mutation may play an important protective role in the development of hepatocellular carcinoma (Minmin et al., 2011). Individuals carrying the rs2737190-AG genotype, compared with AA genotype, had a significantly increased risk for tuberculosis, which was more evident among non-smokers (Wang et al., 2016). The results above indicates the gene polymorphism of TLR4 is associated with several different diseases when working together with some other factors. Combined effect of *P. gingivalis* infection with TLR4 SNPs of rs2737190, rs1927911 on periodontitis in the present study may also prove this thought, however, future study in larger sample size is still necessary.

In our study, the difference of rs11536889 was not found between msCP and nmCP groups, which was different from the studies of Ding et al. (2015) and Fukusaki et al. (2007). Ding et al. (2015) found rs11536889 (G > C) in TLR4 displayed a statistically significant difference in distribution between individuals with moderate periodontitis and severe periodontitis. The distribution of the GG genotype in moderate periodontitis was significantly higher than that in the severe periodontitis group. However, the distribution did not significantly differ between the control group and the periodontitis group, and the significant difference of smoking status was not adjusted in Ding’s research. It seems that the association of rs11526889 with periodontitis might be uncertain yet. Fukusaki et al. (2007) also found that it differed significantly among the younger periodontitis patients, which indicated rs11536889 may be associated with periodontitis. The difference between the above results related to the association of rs11536889 and periodontitis may be caused by different individual conditions, age distribution and sample size. Moreover, the relation of rs7873784 and periodontitis was not found associated with periodontitis in our study either. However, a research reported the distribution of haplotype GCG of rs7873784, rs1927907, and rs1153688 of TLR4 was statistically different between moderate and severe periodontitis in North Chinese population (Ding et al., 2015). Whereas rs7873784 alone didn’t contribute to the severity of periodontitis in that research (Ding et al., 2015). This more or less indicates that multiple gene interactions may also contribute to the progression of periodontal disease. Rs7873784 in TLR4 was significantly associated with T2DM in the Chinese population (Huang et al., 2015). The genotypes GG and CG of rs7873784 protected against the development of T2DM among southern Chinese people. The change of allele in rs7873784 from C to G seems to be a good candidate for T2DM. The homogeneous genotype CC of rs7873784 might be a risk factor for T2DM (Huang et al., 2015). While another study on rs7873784 found no direct association with the incidence of T2DM in German (Kolz et al., 2008). In our study, CC genotype of rs7873784 was relatively rare, which means T2DM may not skew the result. Though as mentioned above, rs1927911 and rs7873784 were reported to be possibly associated with T2DM, and the msCP group in the study had a higher percentage of diabetes although non-significantly. As well known, diabetes is a clear risk factor for severity of periodontitis (Genco & Borgnakke, 2013). To diminish such possible impact of T2DM on the severity of periodontitis, we tried to adjust it during the logistic regression.

At present, researches of SNPs rarely consider the presence of bacterial invasion. Laine et al. (2013) has proposed identifying the presence of bacterial species *Tannerella forsythia* (*T. forsythia*), *P. gingivalis, Aggregatibacter actinomycetemcomitans* (*A. actinomycetemcomitans*), and SNPs TNF -857 and IL-1A -889 as discriminators between periodontitis and non-periodontitis. The result showed it is valuable in modeling the multifactorial and complex nature of periodontitis. Therefore, we decided to investigate the possible associations between SNPs of TLR4 with periodontitis considering the most prevalent periodontal pathogen *P. gingivalis*. Because the detection of *A. actinomycetemcomitans* is low in Chinese patients with either chronic or aggressive periodontitis (Feng et al., 2014; Feng et al., 2009; Li et al., 2015). *T. forsythia* is one of the most common periodontal pathogenic bacteria and it possesses a glycosylated S-layer as an outermost cell decoration, which was so-called LPS. The S-layer provides a selection advantage to the bacterium in the natural habitat, which was benefit for the colonization of other periodontal pathogens. But its virulence potential remains unclear (Yoshimura et al., 2002). Conversely, LPS of *P. gingivalis* may stimulate a few classic pathways that mediate series of inflammation and immune reactions in periodontal tissues as well as in circulation. For example, NF-kB signaling system stimulated by LPS participated in most inflammation response in human body (Wang et al., 2000). Moreover *P. gingivalis* is the most important common pathogens in patients with chronic periodontitis and aggressive periodontitis (Ashimoto et al., 1996; Page & Eke, 2007; Yoshimura et al., 2002). These are the reasons why we chose *P. gingivalis* to investigate in the present study. Recently, Sellers et al. (2016) found that there was no association between the TLR4 SNP rs4986790 (Asp299Gly) and periodontitis when *P. gingivalis* was present. Whereas Gursoy’s study (Gursoy et al., 2016) showed polymorphism of rs4986790 in *P. gingivalis* carriers elevated alveolar bone loss. The controversial results in Caucasians indicate the interaction between *P. gingivalis* and SNPs of TLR4 still deserve further research. While our result may suggest that the TLR4 genetic polymorphisms combined with *P. gingivalis* infection offer a solid explanation for more severe periodontal destruction in Chinese.

The participants of this study were from the cohort of Beijing Hypertension Prevention and Management Institution, who were under regular care for prevention or treatment of chronic disease, especially for hypertension and diabetes. That is why our subjects can’t exclude hypertension or diabetes, for the average age of whom was over 50 years old. Considering that age can contribute to the development of chronic disease including the severity of chronic periodontitis, none/mild chronic periodontitis in the elderly subjects may mean possibility of having lower susceptibility. People among elderly population in a developing country like China with both periodontally and systemically healthy conditions were rare. Therefore, we had to divide the subjects into two groups, none/mild and moderate/severe chronic periodontitis group, to discriminate the severity of periodontal destruction. Both diabetes and smoking are independent risk factors for periodontitis. Between two groups, there was no statistically significant difference of diabetes or hypertension, while smoking with statistical difference. To alleviate such confounding effects as much as possible, age, smoking, diabetes, hypertension all were adjusted during the statistical analysis. Nevertheless, larger samples and functional tests are still necessary to confirm our results in the future.

## Conclusion

Therefore, SNPs of Toll-like receptors 4, rs1927911 (TT/CC+CT), rs2149356 (TT/GG+GT) and rs2737190 (GG/AA+AG) were found associated with severe periodontal destruction in north Chinese periodontitis patients infected with *P. gingivalis*. *P. gingivalis*—TLR4 interaction plays an important role in the pathogenesis of periodontitis.

##  Supplemental Information

10.7717/peerj.7828/supp-1 Supplemental Information 1 TLR4 Data

## Additional Information and Declarations

### Data Availability

The following information was supplied regarding data availability: The raw measurements are available in the Supplemental Files.
